# Income-related inequalities in diagnosed diabetes prevalence among US adults, 2001−2018

**DOI:** 10.1371/journal.pone.0283450

**Published:** 2023-04-13

**Authors:** Yu Chen, Xilin Zhou, Kai McKeever Bullard, Ping Zhang, Giuseppina Imperatore, Deborah B. Rolka

**Affiliations:** Division of Diabetes Translation, Centers for Disease Control and Prevention, Atlanta, GA, United States of America; Governors State University, UNITED STATES

## Abstract

**Aims:**

The overall prevalence of diabetes has increased over the past two decades in the United States, disproportionately affecting low-income populations. We aimed to examine the trends in income-related inequalities in diabetes prevalence and to identify the contributions of determining factors.

**Methods:**

We estimated income-related inequalities in diagnosed diabetes during 2001−2018 among US adults aged 18 years or older using data from the National Health Interview Survey (NHIS). The concentration index was used to measure income-related inequalities in diabetes and was decomposed into contributing factors. We then examined temporal changes in diabetes inequality and contributors to those changes over time.

**Results:**

Results showed that income-related inequalities in diabetes, unfavorable to low-income groups, persisted throughout the study period. The income-related inequalities in diabetes decreased during 2001−2011 and then increased during 2011−2018. Decomposition analysis revealed that income, obesity, physical activity levels, and race/ethnicity were important contributors to inequalities in diabetes at almost all time points. Moreover, changes regarding age and income were identified as the main factors explaining changes in diabetes inequalities over time.

**Conclusions:**

Diabetes was more prevalent in low-income populations. Our study contributes to understanding income-related diabetes inequalities and could help facilitate program development to prevent type 2 diabetes and address modifiable factors to reduce diabetes inequalities.

## 1. Introduction

Diabetes is a health-threatening disease, and its prevalence has been increasing in the United States over the past two decades. The age-adjusted prevalence of diagnosed diabetes among adults aged ≥18 years increased from 6.4% in 1999−2002 to 9.4% in 2013−2016 [1]. It is estimated that 34.1 million adults aged ≥18 years (13% of all US adults) had diabetes in 2018 and 88 million adults had prediabetes, meaning they were at high risk for developing type 2 diabetes [1]. The burden of diabetes falls disproportionately on low-income populations. It was suggested that between 2011 and 2014, compared with persons with high income, the relative percentage increase in diabetes prevalence was 40.0%, 74.1%, and 100.4% for those classified as middle income, near-poor and poor, respectively [2].

Many factors have been identified as contributors to these income disparities in diabetes, including differences in demography (e.g., age and race/ethnicity) and financial resources, differential access to health care services, availability of healthy foods or places to exercise, and differences in health-related behaviors (e.g., smoking) between income groups [3,4]. For example, older adults and racial/ethnic minorities had a higher prevalence of diabetes, and they were more concentrated among low-income groups [4,5]. In addition, risk factors such as obesity and physical inactivity were more prevalent among lower-income populations [6–8]. Possible explanations for the obesity relationship included that people in low-income households were more likely to consume less healthy diets (e.g., nutritionally poor foods) that contributed to weight gain and increased the risk of type 2 diabetes [9]. Lower-income groups also tended to have lower physical activity levels, which were associated with increased insulin resistance and insufficient glycemic control [10]. These risk factors are modifiable and have been particularly important for type 2 diabetes prevention [11].

Although previous studies have evaluated income disparity and risk factors in the prevalence of diabetes in the United States [2,12], there have been no studies that estimated recent changes in income-related inequalities in diabetes and the contributors to these changes over time. The objectives of this study were (1) to examine trends in income-related inequalities in diabetes prevalence during 2001−2018 among US adults; (2) to identify the relative contributions of determining factors to the inequalities; and (3) to quantify the contributions of determining factors to the changes in inequalities over time.

## 2. Research design and methods

### 2.1 Data and study population

Our analysis was based on data from the public-use National Health Interview Survey (NHIS), which is conducted by the Centers for Disease Control and Prevention’s National Center for Health Statistics. NHIS is an annual cross-sectional household survey that is representative of the non-institutionalized civilian US population. NHIS uses a complex multistage sample design to collect data through in-person interviews on sociodemographic and health topics. Our study used 2001−2018 NHIS and included participants aged ≥18 years. Diabetes was identified by the survey question, “Other than during pregnancy, have you ever been told by a doctor or health professional that you have diabetes or sugar diabetes?”

In the decomposition analysis of income-related inequalities in diabetes, we selected determining factors that have been shown to be associated with diabetes and income disparities [4,12], including age (≤44, 45–64, and ≥65), sex, race/ethnicity (Hispanic, non-Hispanic White, non-Hispanic Black, and non-Hispanic other), income-to-poverty ratio, body mass index (BMI) category (underweight/normal, overweight, and obesity), smoking status (former/current smokers and non-smokers), general health status (poor or not), health insurance status (uninsured and insured), and physical activity level (inactivity/low-activity and medium/high activity).

### 2.2 Methods

We assessed income-related inequalities in diabetes prevalence using the Concentration Index (CI) [13]. The CI was derived from the concentration curve, which plotted the cumulative proportion of diabetes against the cumulative proportion of population ranked by income (Appendix Figure S1 in S1 File). The CI was defined as twice the area between the concentration curve and the line of equality (the 45-degree line), which can be expressed as:

$$CI=\frac{2}{nu}\sum_{i=1}^{n}y_{i}R_{i}-1$$ (1)

where *y*_*i*_ was the binary diabetes status for individual *i*, *n* was the sample size, *u* was the mean of *y*_*i*_, and *R*_*i*_ was the fractional rank of individual *i* in the income-to-poverty ratio distribution. The income-to-poverty ratio was used as the measure of income throughout the paper [14], and it is the ratio of a family’s income to the federal poverty threshold. The advantage of using this ratio is that it considers family size and composition and thus is comparable across households. For the binary diabetes status, the CI was normalized by dividing by 1 minus the prevalence of diabetes [15]. The CI ranged from -1 to 1, with 0 indicating complete equality in the distribution of diabetes across income. Negative (positive) values suggested that diabetes was concentrated among lower-income (higher-income) groups. The absolute value of CI measures the degree of inequality with the larger value indicating greater disparity. We estimated the CI overall and by sex, age, and race/ethnicity annually between 2001 and 2018.

Using Wagstaff decomposition [13], we decomposed the overall CI into explanatory variables to examine their separate contributions to the CI. Wagstaff et al. [13] suggested that if the outcome variable *y* (i.e., diabetes status) can be explained linearly using a set of *k* determining factors:

$$y=\alpha +\sum_{k}\beta_{k}x_{k}+\varepsilon$$ (2)

Then we can quantify the contribution of each factor to the CI using the decomposition equation:

$$CI=\sum_{k}(\frac{\beta_{k}{\bar{x}}_{k}}{u}){CI}_{k}+\delta =\sum_{k}\eta_{k}{CI}_{k}+\delta$$ (3)

where *β*_*k*_, ${\bar{x}}_{k}$, and *CI*_*k*_ were the coefficient, mean, and CI of *x*_*k*_ (measured the income-related inequalities in *x*_*k*_), respectively; *u* was the mean of *y*, and *δ* was the residual. Given the binary diabetes status, we applied probit regression with marginal effects (*β*_*k*_) [16]. ∑_*k*_*η*_*k*_*CI*_*k*_ was the contribution of all factors to the CI, which was a weighted sum of the *CI*_*k*_ and the weight was *η*_*k*_ (i.e., the elasticity of *y* with respect to *x*_*k*_). *η*_*k*_ indicated the percentage change in diabetes associated with the percentage change in *x*_*k*_. The contribution of *x*_*k*_ to the CI was calculated as *η*_*k*_*CI*_*k*_/*CI*, with a larger value representing a larger contribution.

After calculating the annual CI and contributions of determining factors to the CI, we examined the contributions of determining factors to the changes in CI over time. We identified the trend in CI using Joinpoint regression [17], and we found that the absolute value of CI (i.e., the degree of diabetes inequality) decreased during 2001−2011 and increased thereafter. Therefore, we decomposed the changes in CI in 2001−2011 and 2011−2018, respectively, following Wagstaff et al. [13]:

$$\Delta CI={CI}_{2}-{CI}_{1}=\sum_{k}\eta_{k2}({CI}_{k2}-{CI}_{k1})+\sum_{k}{CI}_{k1}(\eta_{k2}-\eta_{k1})+\Delta \delta$$ (4)

where Δ*CI* was the difference in CI between two years (i.e., Δ*CI* = *CI*_2011_−*CI*_2001_ during 2001−2011; Δ*CI* = *CI*_2018_−*CI*_2011_ during 2011−2018). The first term denoted the change in CI caused by changes in the *CI*_*k*_. The second term indicated to what extent a change of *η*_*k*_ numerically impacted on Δ*CI*.

The conceptual framework for the analytic approach was presented in Appendix Figure S9 in S1 File. All analyses incorporated sampling weights and accounted for the complex survey design. Data were analyzed using Stata (version 14).

## 3. Results

### 3.1 Trends in income-related inequalities of diabetes

**Fig 1:**
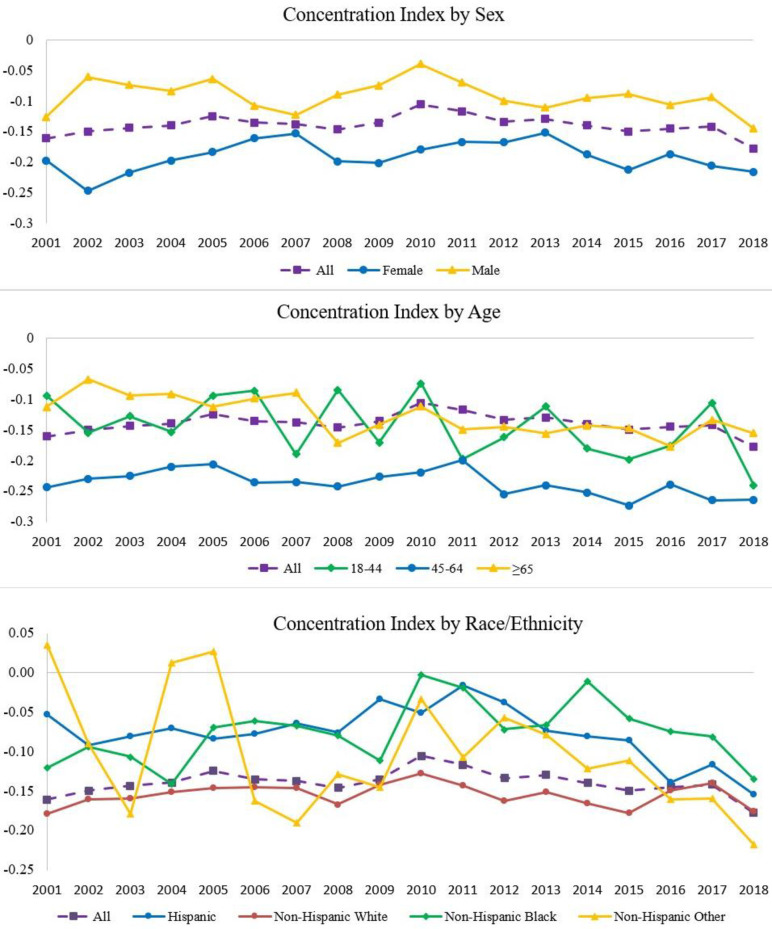
Income-related inequalities in diabetes by sex, age, and race/ethnicity among US adults aged ≥ 18 years, 2001–2018. The concentration index (CI) measures the inequality in diabetes prevalence over the distribution of income. Negative CIs indicate that diabetes was concentrated among lower-income groups, and a larger CI (in absolute value) indicates a greater degree of inequality.

Trends in income-related inequalities of diabetes during 2001−2018 are presented in Fig 1. We found that all CIs were negative, which indicated that diabetes was more concentrated among low-income groups. Overall, the degree of inequality (absolute value of CI) decreased from 0.161 in 2001 to 0.117 in 2011 (annual percentage change [APC] = -2.5, *p* = 0.013, Appendix Table S1 in S1 File), and then increased to 0.177 in 2018 (APC = 4.7, *p* = 0.004). The degree of inequality differed by sex and age group. Females had a higher level of inequality in diabetes than males for all years. For females, the degree of inequality decreased during 2001−2012 (APC = -2.5, *p* = 0.024) and increased after 2012 (APC = 4.7, *p* = 0.097); for males, the trend of the degree of inequality was not statistically significant. Among age groups, middle-aged adults (45−64 years) had the highest level of inequality in diabetes. The degree of inequality increased in each age group during 2001−2018 (all *p*<0.05). In addition, we found that females aged 45–64 years had the highest level of inequality in diabetes (Appendix Figure S2 in S1 File), and the trend of the degree of inequality was not statistically significant. Males aged ≥65 years or 18–44 years had relatively low levels of inequality in diabetes compared to other groups. Diabetes inequality also differed by race/ethnicity. The non-Hispanic White population had higher level of inequality and the degree of inequality was relatively stable during 2001−2018 (Fig 1). For Hispanic and non-Hispanic other populations, the degree of inequality increased during 2012–2018 (*p*<0.05).

### 3.2 Decomposition of inequalities in diabetes

We decomposed annual income-related diabetes inequalities into determining factors and we reported results in 2001, 2011, and 2018 (Table 1). The table presents the elasticities ($\eta_{k}=\frac{\beta_{k}{\bar{x}}_{k}}{u}$), the CI for the determining factors (*CI*_*k*_), and the contributions of determining factors to diabetes inequalities. Income was the leading contributor to inequalities in diabetes, contributing 48%, 62%, and 55% of the inequalities in 2001, 2011 and 2018, respectively.

**Table 1:** Decompositions of income-related inequalities in diabetes in 2001, 2011, and 2018.

	2001				2011				2018			
	*η*_*k*_ *	*CI*_*k*_ †	Contribution‡		*η*_*k*_	*CI*_*k*_	Contribution		*η*_*k*_	*CI*_*k*_	Contribution	
			Absolute	%			Absolute	%			Absolute	%
**Age, years** (Ref: ≤44)												
45–64	0.295	0.218	0.064	-40	0.339	0.215	0.073	-63	0.282	0.159	0.045	-25
≥65	0.295	-0.186	-0.055	34	0.325	-0.029	-0.009	8	0.354	-0.030	-0.010	6
**Sex** (Ref: Female)												
Male	0.052	0.077	0.004	-2	0.057	0.072	0.004	-4	0.063	0.068	0.004	-2
**Race/ethnicity** (Ref: Non-Hispanic White)												
Hispanic	0.036	-0.365	-0.013	8	0.058	-0.341	-0.020	17	0.050	-0.321	-0.016	9
Non-Hispanic Black	0.046	-0.264	-0.012	8	0.039	-0.256	-0.010	9	0.034	-0.263	-0.009	5
Non-Hispanic other	0.016	0.013	0.000	0	0.029	-0.011	0.000	0	0.050	0.016	0.001	0
**Income-to-poverty ratio**	-0.197	0.393	-0.077	48	-0.174	0.415	-0.072	62	-0.238	0.411	-0.098	55
**BMI category**§(Ref: Underweight/normal)												
Overweight	0.124	0.047	0.006	-4	0.127	0.063	0.008	-7	0.133	0.059	0.008	-4
Obesity	0.312	-0.079	-0.025	15	0.363	-0.098	-0.036	31	0.358	-0.114	-0.041	23
**Smoking status** (Ref: Never)												
Former/current smoker	0.097	-0.030	-0.003	2	0.062	-0.070	-0.004	4	0.059	-0.087	-0.005	3
**Physical activity**^||^ (Ref: Medium/high activity)												
Inactivity/low activity	0.103	-0.224	-0.023	14	0.132	-0.219	-0.029	25	0.089	-0.246	-0.022	12
**Health insurance status** (Ref: Insured)												
Uninsured^¶^	-0.050	-0.444	0.022	-14	-0.051	-0.486	0.025	-21	-0.030	-0.429	0.013	-7
**General health status**^**#**^ (Ref: fair to excellent)												
Poor general health	0.058	-0.423	-0.025	15	0.044	-0.398	-0.018	15	0.036	-0.455	-0.016	9
**Residual (Other factors)**			-0.024	15			-0.029	25			-0.031	17
**Overall CI**			-0.161	100			-0.117	100			-0.177	100

* *η*_*k*_ is the unit-free measure of association, namely the percentage change in diabetes associated with the percentage change in the determinant factor.

† The CI of each determinant factor measures the degree of inequality in the prevalence of the determinant factor across the income distribution. This ranges from -1 to +1; its negative values imply that the determinant is concentrated among individuals with lower incomes, while the opposite is true for its positive values. The CIs of determining factors revealed that older adults (aged ≥65 years), Hispanic individuals, non-Hispanic Black individuals, adults with obesity, smokers, adults with low physical activity levels, uninsured persons, and adults with poor general health were concentrated among low-income groups. In contrast, middle-aged adults, males, and overweight adults were concentrated among high-income groups.

‡ “Absolute” contribution is the product of *η*_*k*_ and *CI*_*k*_ as indicated by Eq (3).

§ BMI category: Underweight/normal (BMI <25), overweight (25 ≤ BMI < 30), and having obesity (BMI ≥ 30).

^||^Level of physical activity was categorized as recommended in the 2008 physical activity guidelines (https://health.gov/sites/default/files/2019-09/paguide.pdf): Inactive − Adults engaging in no leisure-time physical activity; Low activity − activity beyond baseline but fewer than 150 minutes of moderate-intensity physical activity a week or the equivalent amount (75 minutes) of vigorous-intensity activity; Medium activity − 150 minutes to 300 minutes of moderate-intensity activity a week (or 75 to 150 minutes of vigorous-intensity physical activity a week, or an equivalent combination of moderate- and vigorous-intensity aerobic activity); High activity − Moderate-intensity for > 300 minutes/week, or vigorous-intensity for > 150 minutes/week, or an equivalent combination).

¶ The uninsured are persons who did not report having health insurance at the time of the interview under private health insurance, Medicare, Medicaid, State Children’s Health Insurance Program (CHIP), a State-sponsored health plan, other government programs, or military health plan (includes TRICARE, VA, and CHAMP-VA). This definition of uninsured matches that used in the Health United States.

# Self-reported general health status that comes from the survey question: “Would you say your health in general is excellent, very good, good, fair, or poor?”

**Fig 2:**
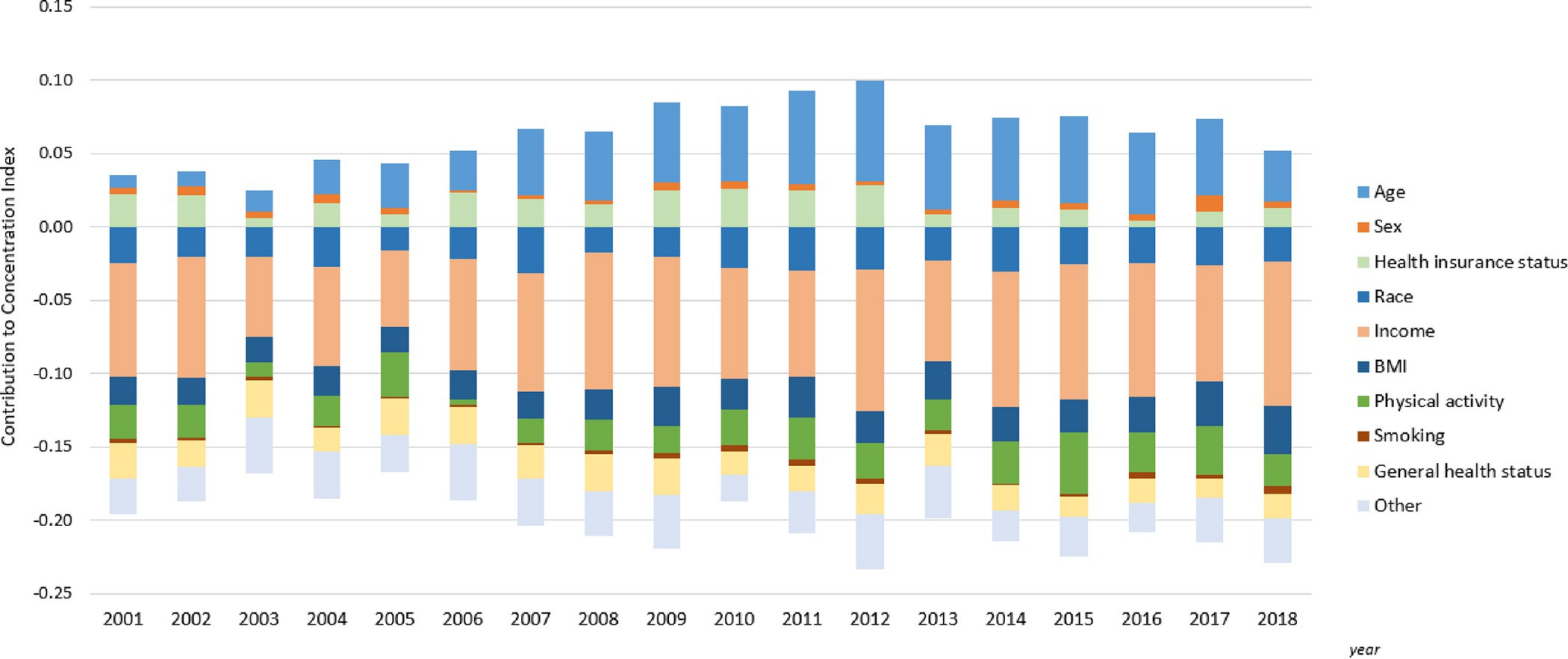
Relative contributions of determining factors to income-related inequalities in diagnosed diabetes, 2001−2018. For each year, the length of the sub-bar indicated the numeric value of each factor’s relative contribution to the overall CI, and the sum of numeric values from all factors was equal to the overall CI.

The contribution of determining factor to diabetes inequality can be read as follows: for example, in 2001, the elasticity for obesity was 0.312 (*η*_*k*_ column), suggesting that a 1% increase in the prevalence of obesity led to a 0.312% increase in the diabetes prevalence (the components of elasticity: *β*_*k*_ and ${\bar{x}}_{k}$ were reported in the Appendix Table S2 in S1 File). Adults with obesity were concentrated in the low-income groups (*CI*_*k*_ = -0.079). By multiplying *η*_*k*_ and *CI*_*k*_, the absolute contribution of obesity to diabetes inequality was -0.025 (Absolute column), thus constituting 15% (Contribution % column) of the overall CI of -0.161 (the bottom of Contribution columns). The negative contributions, such as the contribution of overweight was -4%, suggesting that the extent of diabetes inequality would have been 4% more (closer to -1) if adults with overweight were equally distributed across income. In 2001, income (48%), older adults (age ≥65 years; 34%), Hispanic and non-Hispanic Black individuals (16%), obesity (15%), physical inactivity/low-activity (14%), smoking (2%), and poor general health (15%) contributed to higher diabetes prevalence among low-income groups, while the 45−64 age group (-40%), males (-2%), overweight (-4%) and uninsured status (-14%) counteracted such contributions. Similar patterns were observed in 2011 and 2018. Fig 2 presented the changes in the relative contributions of factors to diabetes inequalities from 2001 to 2018. We observed variations of contributions of each factor over time. Income was always the main contributor to diabetes inequality and age mainly counteracted such contributions. The contributions of income changed with the highest contribution in 2012 and the lowest contribution in 2003. The contributions of age increased during 2001–2011 and decreased thereafter.

### 3.3 Decomposition of changes in inequalities in diabetes

We investigated the changes in diabetes inequality in 2001−2011 and 2011−2018 (Table 2). During 2001–2011, the degree of diabetes inequality decreased, with CI changing from -0.161 to -0.117 (Table 1). Income accounted for 12% of the change in diabetes inequality, which mostly stemmed from a decrease in diabetes-income elasticity. Because average income and the association of income with diabetes (*β*_*k*_) stayed relatively constant, the increase in diabetes prevalence during 2001–2011 (Appendix Figure S3 in S1 File) explained the change in the diabetes-income elasticities. The main contributor to the decrease in inequality during 2001–2011 were older adults (aged≥65 years). Older adults accounted for the largest part (104%) of the change in diabetes inequality, which can be explained by the fact that older adults were less concentrated in the low-income groups in 2011 than they were in 2001 (CI was -0.186 in 2001 and -0.029 in 2011; Table 1). People aged 45–64 years contributed 20% to the change in inequality, which can be derived from an increased share of this age group (from 30.7% to 34.9%; Appendix Table S2 in S1 File) and by reinforcement of the association between age and diabetes (*β*_*k*_ increased from 0.062 to 0.087). Obesity contributed -25% to the change in diabetes inequality. Obesity was more concentrated in the low-income groups in 2011 than it was in 2001. Also, there was an increase in diabetes-obesity elasticity, which in turn derived from an increase in the obesity prevalence and by a reinforcement of the association between obesity and diabetes. Hispanic individuals contributed -16% to the change in inequality due to the increased share of this group and the reinforcement of their association with diabetes. Physical inactivity/low activity contributed -11% to the change, mainly because of the reinforcement of the association between physical inactivity/low activity and diabetes.

**Table 2:** Decomposition of changes in diabetes inequalities during 2001−2011 and 2011−2018.

	Change 2001−2011				Change 2011−2018			
	ΔE*	ΔC	Total		ΔE	ΔC	Total	
			Absolute	%			Absolute	%
**Age, years** (Ref: ≤ 44)								
45–64	0.010	-0.001	0.009	20	-0.012	-0.016	-0.028	47
≥65	-0.006	0.051	0.046	104	-0.001	0.000	-0.001	2
**Sex** (Ref: Female)								
Male	0.000	0.000	0.000	0	0.000	0.000	0.000	0
**Race/ethnicity** (Ref: Non-Hispanic White)								
Hispanic	-0.008	0.001	-0.007	-16	0.002	0.001	0.003	-5
Non-Hispanic Black	0.002	0.000	0.002	5	0.001	0.000	0.001	-2
Non-Hispanic other	0.000	-0.001	-0.001	-1	0.000	0.001	0.001	-2
**Income-to-poverty ratio**	0.009	-0.004	0.005	12	-0.027	0.001	-0.026	43
**BMI category** (Ref: Underweight/normal)								
Overweight	0.000	0.002	0.002	5	0.000	0.000	0.000	0
Obesity	-0.004	-0.007	-0.011	-25	0.001	-0.006	-0.005	8
**Smoking status** (Ref: Never)								
Former/current smoker	0.001	-0.002	-0.001	-3	0.000	-0.001	-0.001	1
**Physical activity** (Ref: Medium/high activity)								
Inactivity/low activity	-0.006	0.001	-0.005	-11	0.009	-0.002	0.007	-12
**Health insurance status** (Ref: Insured)								
Uninsured	0.000	0.002	0.002	5	-0.010	-0.002	-0.012	20
**General health status** (Ref: Fair to excellent)								
Poor general health	0.006	0.001	0.007	16	0.003	-0.002	0.001	-2
**Residual**			-0.005	-10			-0.001	1
**Overall CI**			0.044	100			-0.060	100

*ΔE equals $\sum_{k}{CI}_{k1}(\eta_{k2}-\eta_{k1})$ and ΔC equals $\sum_{k}\eta_{k2}({CI}_{k2}-{CI}_{k1})$ as shown in Eq (3).

The degree of diabetes inequality increased from 2011 to 2018, with CI changing from -0.117 to -0.177. The main contributors to the change were income (43%) and the 45−64 age group (47%). The contribution of income could be derived from an increase in average income and by the strengthening of the negative association between income and diabetes. The contribution of the 45−64 age group can be mainly explained by the fact that this age group was less concentrated in the higher-income groups in 2018 than it was in 2011, with CI decreasing from 0.215 in 2011 to 0.159 in 2018. People aged ≥65 years contributed 2% to the change in diabetes inequality, which stemmed from an increased proportion of this age group and by reinforcement of the association with diabetes. Uninsured status contributed 20% to the change in diabetes inequality, which could stem from a decreased proportion of the uninsured population. Obesity contributed 8% to the change in inequality, and the contribution was mainly because adults with obesity were more concentrated in the low-income groups over this period (CI changed from -0.098 to -0.114). Physical inactivity/low activity contributed -12% to the change in inequality, which was mainly due to the lower prevalence of physical inactivity/low activity and its weaker association with diabetes in 2018 than in 2011.

## 4. Discussion

Our study contributes to understanding socioeconomic disparities in diabetes, which could be beneficial for developing programs to provide care to people in lower socioeconomic positions. Our results showed that: first, diabetes was more prevalent in low-income populations during 2001−2018. Income was the most important contributor to diabetes inequalities, after accounting for other individual-level factors. Moreover, obesity, physical activity, and race/ethnicity were the main contributors to diabetes inequalities at almost all time points. Second, the income-related inequalities in diabetes were larger among females and middle-aged adults (aged 45–64 years). Third, the degree of income-related inequality in diabetes decreased from 2001 to 2011 and have become widened from 2011 to 2018.

Previous studies [2,18] showed that socioeconomic disparities existed in diabetes prevalence and that the prevalence declined as income increased. Our decomposition analysis revealed that income was the leading contributor to diabetes inequalities, accounting for 48%, 62%, and 55% of the inequalities in 2001, 2011 and 2018, respectively. The finding was consistent with previous findings that income accounted for a large share of health inequalities [19,20]. One possible explanation is that sufficient income provides more opportunities for essential preventive services, such as prediabetes screening and lifestyle intervention services. Income disparity also creates differences in other health determinants, for example, low income is associated with food insecurity and lower diet quality [21]. Also, low-income households are likely to have a poor living environment (e.g., lack of recreational areas that discourage physical activity, and lack of supermarkets limits the access to healthy foods) which increases the risk of diabetes [12,18]. Obesity and lifestyle factors are also important contributors to income-related diabetes inequalities. As previous studies suggested [6,7], we found that obesity and physical inactivity/low activity were associated with diabetes and were more prevalent among lower-income populations. In a national sample of adults, it was estimated that for every 1-kilogram increase in weight, the diabetes prevalence increased by 4.5% [6]. Physical inactivity is associated with increased insulin resistance and poorer glycemic control independent of body weight [10]. Lower-income groups are more likely to have barriers to physical activity because of cost, time constraints, and lack of facility accessibility [22]. Moreover, racial/ethnic minorities have higher diabetes prevalence, and socioeconomic factors are important contributors to these disparities [4]. Our results showed that racial/ethnic minorities were associated with income-related diabetes inequalities. Previous studies found that Hispanic and non-Hispanic Black individuals have greater rates of poverty than the non-Hispanic White population, and risk factors of diabetes such as obesity, food insecurity, and low physical activity are more prevalent in racial/ethnic minorities [4,23]. Strategies focused on improving access and quality of care are suggested to reduce racial and ethnic disparities in diabetes [24].

We observed greater income-related inequalities in diabetes among females and middle-aged adults (aged 45–64 years). Gender and age differences were also evident in the contributors to diabetes inequalities. The decomposition results by gender (Appendix Figures S4 and S5 in S1 File) showed that BMI was more influential for females than males, which was mainly because obesity was more concentrated among low-income groups for females. Females tend to experience unfavorable socioeconomic circumstances than males [25], and low-income females are more likely to develop obesity and diabetes which are influenced by psychological and other risk factors linked to poverty [26,27]. The results also showed that smoking was more influential for males than females. Consistent with prior studies [28], we found that the smoking rate was higher among males than females, especially among the low-income population. The decomposition results by age group (Appendix Figures S6-S8 in S1 File) showed that the pattern of factors’ contributions was relatively consistent over time for middle-aged adults and older adults. Whereas for adults aged≤44 years, the factors’ contributions showed more variations over time. Compared to older adults, we found that BMI was more influential for middle-aged adults. This is mainly because the obesity rate was higher for middle-aged adults than older adults [29], especially among low-income groups. On the contrary, physical inactivity was more influential for older adults than other age groups, given that older adults had a higher prevalence of physical inactivity than others [30]. These differences implied that clinical and public health strategies on preventing diabetes could be adapted to specific gender and age groups.

Another notable finding is that income-related inequalities in diabetes appear to have widened over the past decade. During 2001−2011, diabetes prevalence increased relatively more among the economically better off than other income groups (Appendix Figure S3 in S1 File), reducing the disparities in diabetes prevalence across income groups. However, starting from 2011, the lowest income group had a faster increase in diabetes prevalence, while prevalence was relatively stable among high-income groups. Correspondingly, we observed a widening diabetes inequality in diabetes prevalence. Income accounted for 43% of the change in diabetes inequality, which could be associated with the strengthening of the negative association between income and diabetes during 2011–2018 and the increase in average income over time. The widened diabetes inequality could also be partly explained by the changes in the age structure across income groups over time. Specifically, the proportion of middle-aged adults increased in lower-income groups during 2011 and 2018 and this age group contributed to the increase in diabetes in the lower-income group. One possible explanation is that this age group was impacted by the 2008 financial crisis and experienced a high long-term unemployment rate and loss of income. The financial difficulties for middle-aged adults could also increase their risk of diabetes. Another possible explanation for the increased diabetes prevalence among lower-income groups is that the passage of the Affordable Care Act (ACA) in 2010 provided access to care to the previously uninsured population. A major provision of the ACA was to expand Medicaid, through which low-income populations got access to medical care and received preventive services, including diabetes screening [31]. Kaufman et al. [32] demonstrated that Medicaid expansion increased the size of the low-income population with newly identified diabetes, which may explain the increased diabetes prevalence (Appendix Figure S3 in S1 File) and the decreased proportion of the uninsured population starting from 2011 (Appendix Table S2 in S1 File).

The widening income-related diabetes inequality in the past decade also highlights the urgent need for reducing disparity in diabetes across income groups. Improving income level of the low-income group through policy interventions such as providing more employment opportunities and increasing high school and college graduation rate could reduce the income-related diabetes inequality. Programs that can reduce diabetes-related risk factors of type 2 diabetes among lower-income populations could also help to close the gap in diabetes between income groups. Low-income populations were more likely to consume nutritionally poor diets and have lower physical activities levels [9,10]. Implementing effective programs and interventions that can improve the access to fruit and vegetable through subsidies and increasing the level of physical activities by improving neighborhood safety and living environment among low-income populations may reduce income-related inequality in diabetes. Scaling up the National Diabetes Prevention Program among those who are at high risk of developing type 2 diabetes in low-income communities could also reduce diabetes incidence in the low-income population and close the gap in diabetes between income groups.

Our study was subject to several limitations. First, self-reported data are subject to recall and social desirability bias. However, self-reported diagnosed diabetes was shown to have high reliability [33]. We used an imputed income-to-poverty ratio which could have a biased effect on the imputed values if income was misreported. Second, we focused on diagnosed diabetes, and findings did not reflect disparities in the prevalence of all diabetes; approximately 28% of all diabetes is undiagnosed [33]. Third, our cross-sectional study described the observed patterns of income-related inequalities in diabetes, and the decompositions were not able to provide causal pathways between diabetes inequality and its determining factors. Given that diabetes usually develops slowly and income changes over time, future studies may explore the causal relationship between diabetes and income. Fourth, although area-level social determinants of health (SDH) were well-understood to influence diabetes disparities [34], we were not able to analyze the area-level SDH due to the limitation of public-use NHIS data. Fifth, populations such as Asian American and Pacific Islander, multiple-race and American Indian and Alaskan Native were classified as “Non-Hispanic other” race/ethnicity group in NHIS due to small sample sizes and disclosure risk.

Socioeconomic inequalities in diabetes have persisted over the past two decades in the United States, with lower-income populations being more affected. By exploring income-related inequalities in diabetes from 2001 to 2018, we found such diabetes inequalities narrowed in 2001−2011 and appeared to widen in 2011−2018. Monitoring socioeconomic inequality in diabetes proactively could be important to inform policies that reduce the burden of diabetes. Additionally, developing and scaling effective diabetes prevention interventions among lower-income populations could also help reduce diabetes inequalities.

## Supporting information

S1 File (DOCX)
