# Supplementary material for: Income-related inequalities in diagnosed diabetes prevalence among US adults, 2001−2018
Source: PLoS One. 2023 Apr 13;18(4):e0283450. doi: 10.1371/journal.pone.0283450 (PMC10101461; doi:10.1371/journal.pone.0283450)
Supplement: S1 File — (DOCX) [file pone.0283450.s001.docx]

**Appendix A:** Calculation of Concentration Index (CI)

The concentration index (CI) was derived from the concentration curve (labeled *C* in Figure S1 below), which plotted the cumulative proportion of $y$ (on the vertical axis; $y$ indicated diabetes in our study) against the cumulative proportion of the population (on the horizontal axis) ranked by income (from lowest income to highest income). The value of CI ranged from -1 to 1, with 0 indicating perfect equality in the distribution of diabetes across income. If *C* lies above the diagonal, diabetes prevalence was larger among low-income groups. The further *C* lies from the diagonal, the greater the degree of inequality in diabetes across the income distribution.

The $CI$ was defined as twice the area between *C* and the diagonal. Following Adam Wagstaff et al. (2003), the *CI* was expressed as:

$$CI=\frac{2cov(y_{i}{, R}_{i})}{u}=\frac{2}{nu}\sum_{i=1}^{n} y_{i}R_{i}-1$$

For a binary health outcome (whether the individual had diabetes or not), the CI was normalized by dividing by 1 minus the prevalence of diabetes (Adam Wagstaff, 2005):

$$CI=\frac{1}{1-u}(\frac{2}{nu}\sum_{i=1}^{n} y_{i}R_{i}-1)$$

where $y_{i}$ was the diabetes status for individual $i$, $u$ was the mean of $y_{i}$, $n$ was the sample size, and $R_{i}$ was the fractional rank of individual $i$ in the income distribution.

C

100%

100%

0%

Cumulative percent of y

Cumulative percent of people, ranked by income

**Figure S1 – Concentration curve.**

Negative (positive) CI values suggested that diabetes was concentrated among lower-income (higher-income) groups. The absolute value of CI measures the degree of inequality with the larger value indicating greater disparity. Multiplying the absolute value of CI by 75 gives the percentage of diabetes that would need to be redistributed from the poorer half to the richer half of the population to arrive at a distribution with a CI value of zero (Koolman & Van Doorslaer, 2004). Given that CI is standardized between -1 and +1, which makes it is possible to compare the direction and magnitude of inequality through time and between populations.

As a measure of income, we used the income-to-poverty ratio provided from the publicly available NHIS imputed income files (for example, 2018 data release in <https://www.cdc.gov/nchs/nhis/nhis_2018_data_release.htm> ). We referred to the income-to-poverty ratio as income throughout our paper. The income-to-poverty ratio is the ratio of a family’s income to the applicable Federal poverty threshold that the Census Bureau defines based on the family’s size. The advantage of using the income-to-poverty ratio is that it considers family size and composition and thus is comparable across households.

**Appendix B:**

**
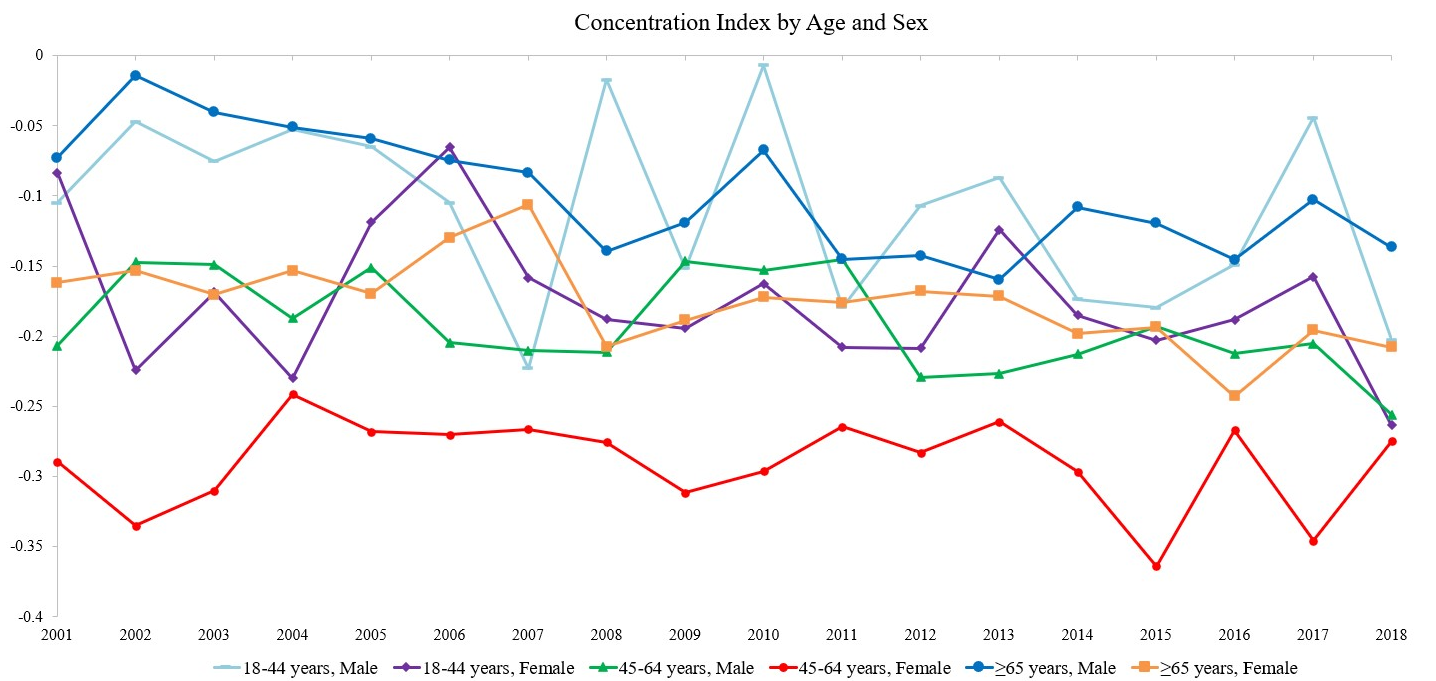
**

**Figure S2 –**Income-related inequalities in diabetes by age and sex among US adults aged ≥ 18 years, 2001-2018**.** The concentration index (CI) measures the inequality in diabetes prevalence over the distribution of income. Negative CIs indicate that diabetes was concentrated among lower-income groups, and a larger CI (in absolute value) indicates a greater degree of inequality.

**Table S1** – **Trend analysis of the degree of income-related inequalities in diabetes using Joinpoint regression*****, 2001−2018.**

|  | Trend | | | |
| --- | --- | --- | --- | --- |
|  | | Years | APC† | *p-value* |
| **Overall** | | 2001-2011 | -2.5 | 0.013 |
|  | | 2011-2018 | 4.7 | 0.004 |
| **Sex** | |  |  |  |
| Female | | 2001-2012 | -2.5 | 0.024 |
|  | | 2012-2018 | 4.7 | 0.097 |
| Male | | 2001-2018 | 1.3 | 0.257 |
| **Age, years** | |  |  |  |
| ≤44 | | 2001-2018 | 3.0 | 0.020 |
| 45-64 | | 2001-2018 | 1.0 | 0.011 |
| ≥65 | | 2001-2018 | 2.8 | 0.003 |
| **Race/ethnicity** | |  |  |  |
| Non-Hispanic White | | 2001-2018 | 0 | 0.941 |
| Hispanic | | 2001-2012 | -3.5 | 0.229 |
|  | | 2012-2018 | 18.9 | 0.001 |
| Non-Hispanic Black | | 2001-2018 | -1.5 | 0.271 |
| Non-Hispanic other‡ | | 2006-2012 | -14.9 | 0.017 |
|  | | 2012-2018 | 19.0 | 0.009 |
| **Age by Sex groups** | |  |  |  |
| 18-44, Male | | 2001-2018 | 3.7 | 0.042 |
| 18-44, Female | | 2001-2018 | 0.9 | 0.381 |
| 45-64, Male | | 2001-2018 | 1.7 | 0.026 |
| 45-64, female | | 2001-2018 | 0.4 | 0.451 |
| ≥65, Male | | 2001-2018 | 3.3 | 0.042 |
| ≥65, female | | 2001-2018 | 2.0 | 0.002 |

*The annual estimates of the Concentration Index were based on sampling surveys which were subjective to sampling variations. These point estimates could over-interpret the results and not represent the population trend. Therefore, we used Joinpoint regression to smooth over these annual estimates and identify the underlying population trend, the change points of the trends, and whether the trend was statistically significant. The number of change points in the Joinpoint regression analyses is determined based on the number of data points. Joinpoint regressions perform multiple tests to recommend the best number of change points, based on this, we identify the trends of overall CI and CIs by subgroups, respectively. †APC: annual percentage change. ‡Among non-Hispanic other populations, the Concentration Index (CI) was positive in 2001, 2004, and 2005 which indicates that diabetes was concentrated among higher-income groups in these years. From 2006, the CIs were negative which indicates that diabetes was concentrated among lower-income groups. We assessed the trend of the degree of income-related inequalities in diabetes among non-Hispanic other populations from 2006 to 2018.

**Table S2** – **The mean values of determinants (**${\bar{\boldsymbol{x}}}_{\boldsymbol{k}}$**) and their associations with diabetes (**$\boldsymbol{\beta}_{\boldsymbol{k}}$**) in the decomposition of diabetes inequalities in 2001, 2011, and 2018.**

|  | 2001 | | 2011 | | 2018 | |
| --- | --- | --- | --- | --- | --- | --- |
|  | $\bar{x}_{k}$* | $\beta_{k}$† | $\bar{x}_{k}$ | $\beta_{k}$ | $\bar{x}_{k}$ | $\beta_{k}$ |
| **Age, years**  (Ref: ≤ 44) |  |  |  |  |  |  |
| 45-64 | 30.7 | 0.062 | 34.9 | 0.087 | 33.3 | 0.088 |
| ≥65 | 16.1 | 0.118 | 17.2 | 0.171 | 20.6 | 0.178 |
| **Male** (Ref: Female) | 48.0 | 0.007 | 48.4 | 0.011 | 48.3 | 0.013 |
| **Race/ethnicity**  (Ref: Non-Hispanic White) |  |  |  |  |  |  |
| Hispanic | 10.8 | 0.022 | 14.2 | 0.037 | 16.3 | 0.032 |
| Non-Hispanic Black | 11.3 | 0.026 | 11.8 | 0.030 | 12.3 | 0.028 |
| Non-Hispanic Other | 4.4 | 0.023 | 5.7 | 0.045 | 7.6 | 0.069 |
| **Income-to-poverty ratio** | 3.9 | -0.003 | 3.8 | -0.004 | 4.2 | -0.006 |
| **BMI category**  (Ref: Underweight/normal) |  |  |  |  |  |  |
| Overweight | 35.6 | 0.022 | 34.5 | 0.033 | 32.5 | 0.040 |
| Obesity | 22.5 | 0.090 | 28.3 | 0.116 | 34.4 | 0.118 |
| **Smoking status**  (Ref: Never) |  |  |  |  |  |  |
| Former/current smoker | 45.0 | 0.014 | 40.9 | 0.014 | 35.9 | 0.017 |
| **Physical activity**  (Ref: Median/high activity) |  |  |  |  |  |  |
| Inactivity/low activity | 56.9 | 0.012 | 51.6 | 0.023 | 46.7 | 0.020 |
| **Uninsured** | 14.7 | -0.022 | 17.3 | -0.027 | 10.4 | -0.030 |
| **Poor general health** | 3.1 | 0.122 | 3.1 | 0.129 | 2.9 | 0.131 |

*Data presented as prevalence (%), except for income-to-poverty ratio, which was presented as mean values. †Marginal effects from the probit model.

**
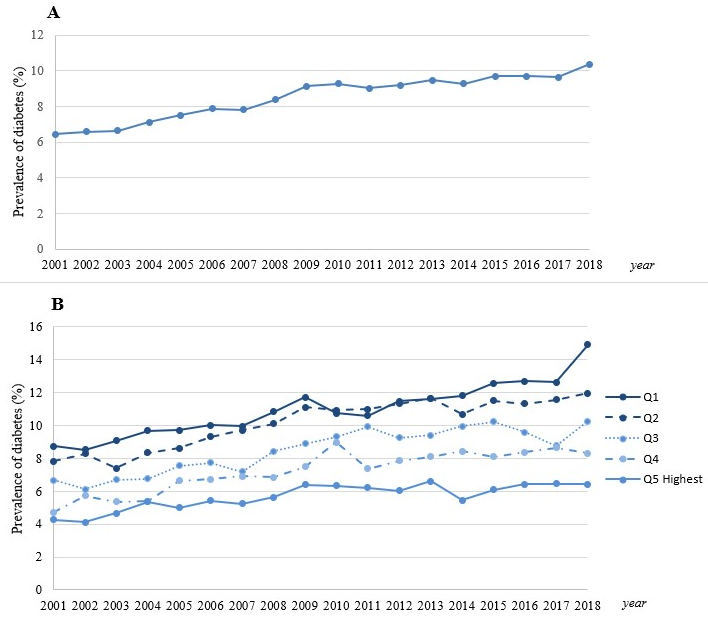
**

**Figure S3 – (A) Trends in prevalence of diagnosed diabetes among US adults aged ≥ 18 years, 2001-2018. (B) Trends in prevalence of diagnosed diabetes by income quantiles among US adults aged ≥ 18 years, 2001-2018. Q=income quantile.**

**Table S3 – Contributions**^*^ **of determining factors to income-related inequalities in diagnosed diabetes, 2001−2018.**

| **Year** | **Age** | **Sex** | **Race** | **Income** | **Physical activity** | **BMI** | **Smoking** | **General health status** | **Health insurance status** | **Other** | **Overall CI** † |
| --- | --- | --- | --- | --- | --- | --- | --- | --- | --- | --- | --- |
| 2001 | 0.009 | 0.004 | -0.025 | -0.077 | -0.023 | -0.019 | -0.003 | -0.025 | 0.022 | -0.024 | -0.161 |
| 2002 | 0.010 | 0.006 | -0.020 | -0.083 | -0.023 | -0.018 | -0.002 | -0.018 | 0.021 | -0.023 | -0.150 |
| 2003 | 0.014 | 0.005 | -0.020 | -0.055 | -0.010 | -0.017 | -0.003 | -0.025 | 0.005 | -0.038 | -0.143 |
| 2004 | 0.024 | 0.006 | -0.028 | -0.067 | -0.021 | -0.021 | -0.001 | -0.017 | 0.016 | -0.032 | -0.139 |
| 2005 | 0.031 | 0.004 | -0.016 | -0.052 | -0.031 | -0.017 | -0.001 | -0.025 | 0.009 | -0.026 | -0.124 |
| 2006 | 0.027 | 0.002 | -0.022 | -0.076 | -0.004 | -0.020 | -0.002 | -0.025 | 0.023 | -0.039 | -0.135 |
| 2007 | 0.045 | 0.003 | -0.032 | -0.080 | -0.016 | -0.018 | -0.002 | -0.022 | 0.019 | -0.032 | -0.137 |
| 2008 | 0.047 | 0.003 | -0.018 | -0.093 | -0.021 | -0.022 | -0.002 | -0.026 | 0.015 | -0.030 | -0.146 |
| 2009 | 0.055 | 0.005 | -0.021 | -0.089 | -0.019 | -0.026 | -0.003 | -0.025 | 0.025 | -0.037 | -0.135 |
| 2010 | 0.051 | 0.006 | -0.028 | -0.076 | -0.025 | -0.021 | -0.004 | -0.016 | 0.025 | -0.018 | -0.105 |
| 2011 | 0.064 | 0.004 | -0.030 | -0.072 | -0.029 | -0.028 | -0.004 | -0.018 | 0.025 | -0.029 | -0.117 |
| 2012 | 0.069 | 0.003 | -0.029 | -0.097 | -0.024 | -0.022 | -0.003 | -0.021 | 0.028 | -0.037 | -0.134 |
| 2013 | 0.057 | 0.003 | -0.023 | -0.068 | -0.021 | -0.027 | -0.002 | -0.021 | 0.009 | -0.036 | -0.129 |
| 2014 | 0.057 | 0.005 | -0.031 | -0.092 | -0.029 | -0.024 | -0.001 | -0.017 | 0.013 | -0.021 | -0.140 |
| 2015 | 0.059 | 0.004 | -0.026 | -0.092 | -0.042 | -0.023 | -0.001 | -0.014 | 0.012 | -0.027 | -0.149 |
| 2016 | 0.055 | 0.005 | -0.025 | -0.091 | -0.026 | -0.024 | -0.004 | -0.017 | 0.004 | -0.020 | -0.145 |
| 2017 | 0.052 | 0.011 | -0.026 | -0.079 | -0.033 | -0.030 | -0.003 | -0.012 | 0.010 | -0.031 | -0.142 |
| 2018 | 0.034 | 0.004 | -0.024 | -0.098 | -0.022 | -0.033 | -0.005 | -0.016 | 0.013 | -0.031 | -0.177 |

* The values in the tables were the absolute contribution of each factor to the overall CI, and the sum value of factors was equal to the overall CI. We can calculate the contribution (%) of each factor to the overall CI through dividing the values in the table by the overall CI, with a larger value representing a larger contribution.

† The overall CI values were the overall annual concentration index.


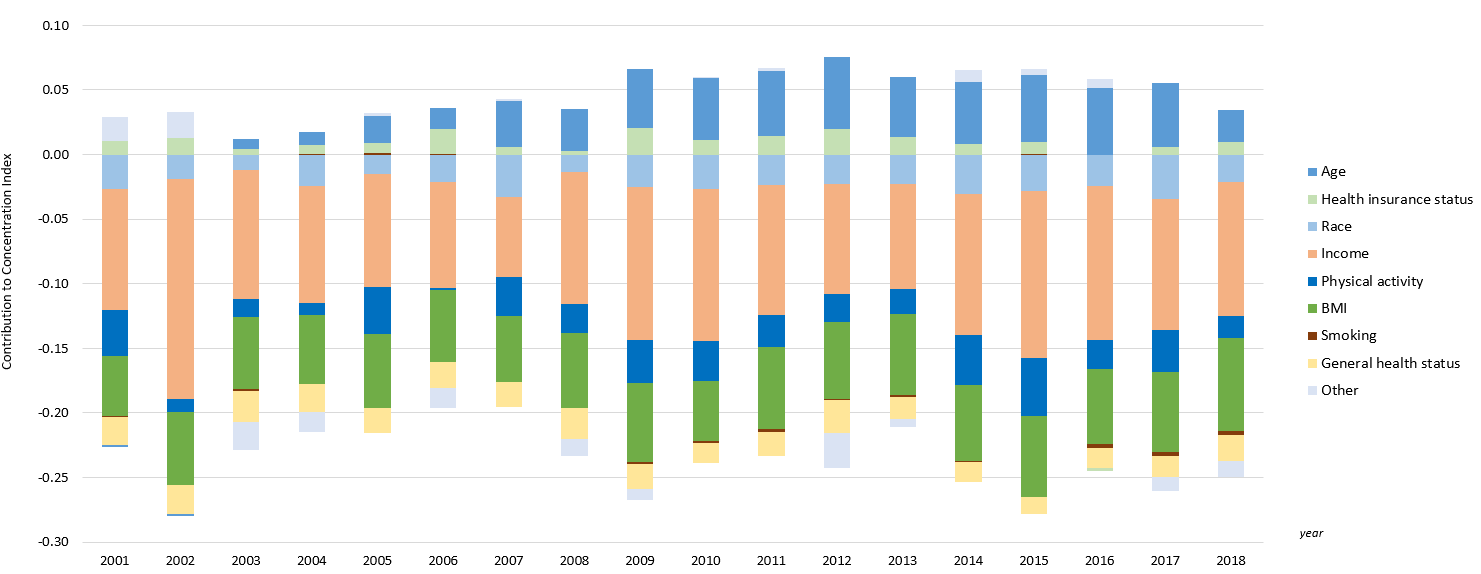


**Figure S4 -** Relative contributions of determining factors to income-related inequalities in diagnosed diabetes among females, 2001−2018.


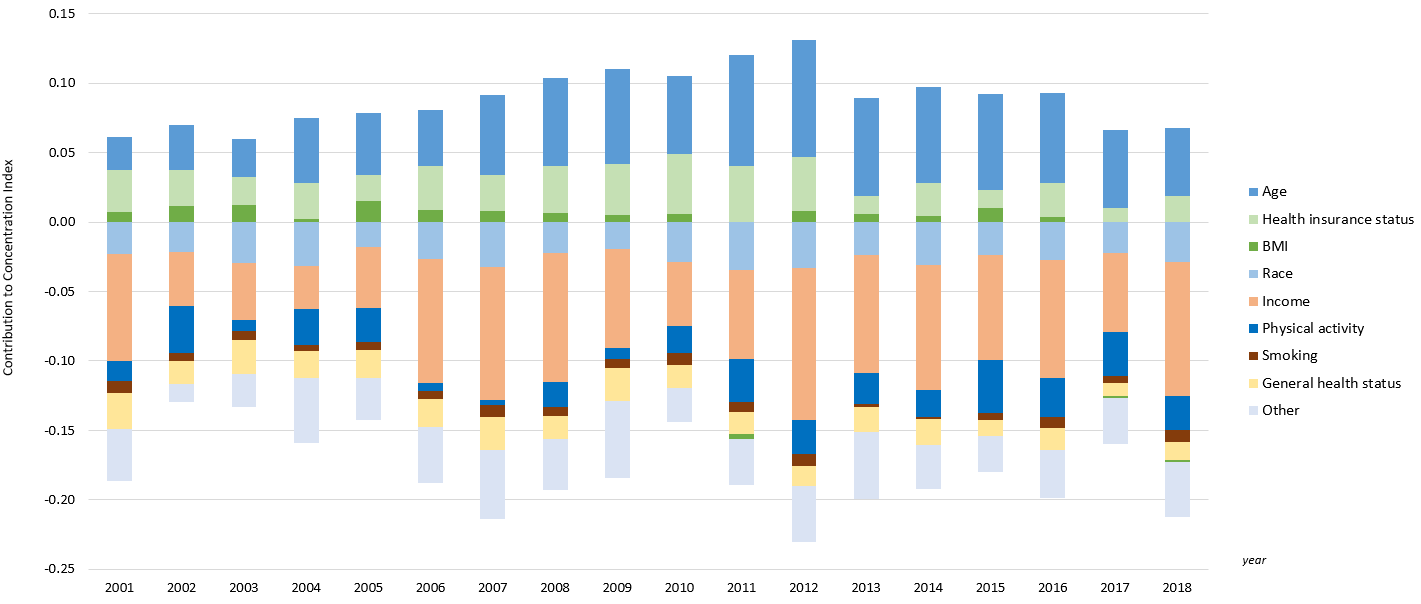


**Figure S5 -** Relative contributions of determining factors to income-related inequalities in diagnosed diabetes among males, 2001−2018.


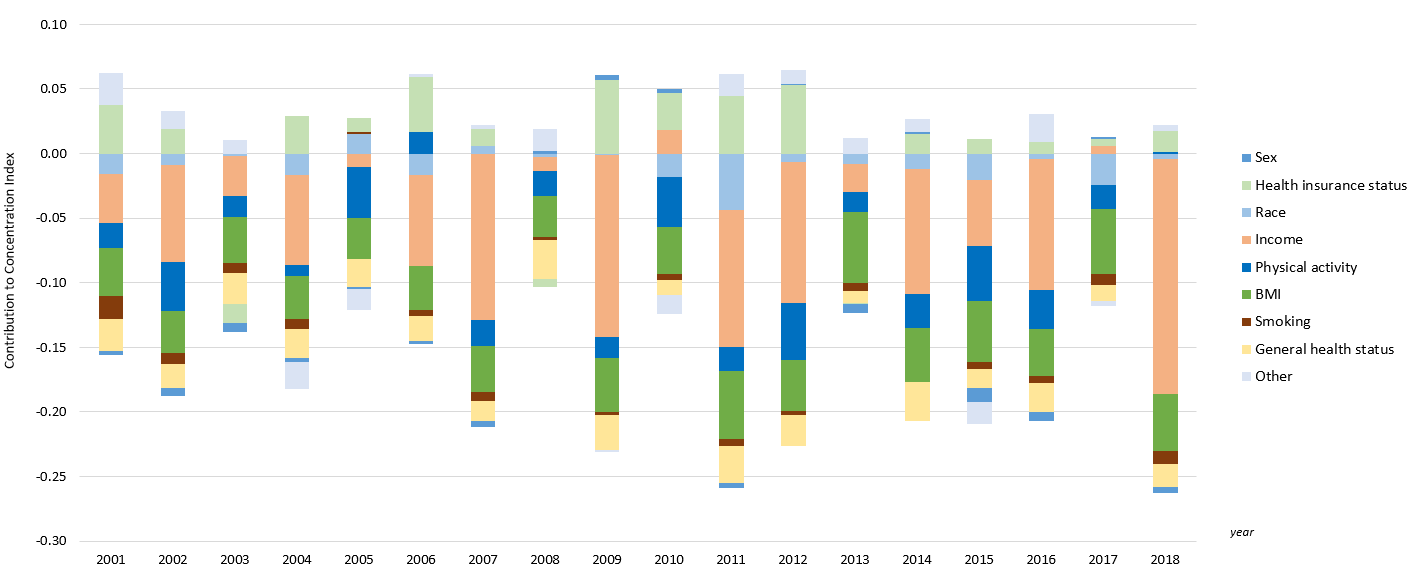


**Figure S6 -** Relative contributions of determining factors to income-related inequalities in diagnosed diabetes among adults aged ≤ 44 years, 2001−2018.


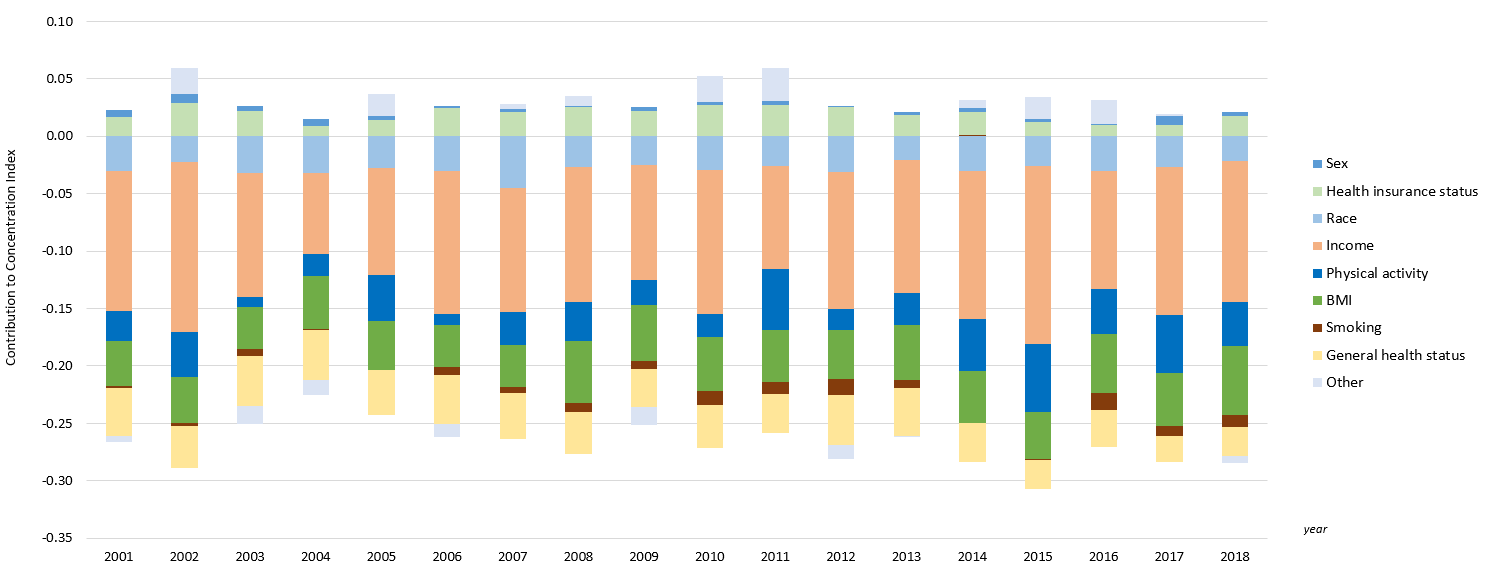


**Figure S7 -** Relative contributions of determining factors to income-related inequalities in diagnosed diabetes among adults aged 45-64 years, 2001−2018.


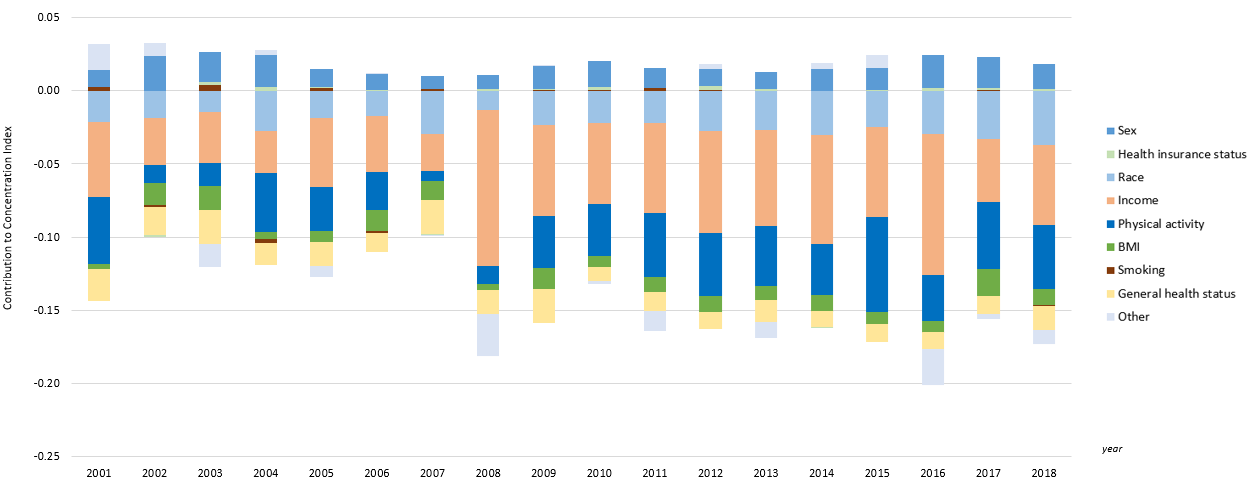


**Figure S8 -** Relative contributions of determining factors to income-related inequalities in diagnosed diabetes among adults aged ≥65 years, 2001−2018.

**1. Income-related inequalities in diabetes:** using Concentration Index (CI):

$CI=\frac{2}{nu}\sum_{i=1}^{n} y_{i}R_{i}-1$

$y_{i}$ was the binary diabetes status for individual $i$, $n$ was the sample size, $u$ was the mean of $y_{i}$, and $R_{i}$ was the fractional rank of individual $i$ in the income-to-poverty ratio distribution.

**2. Wagstaff decomposition:** decomposed the annual overall CI into explanatory variables ($x_{k}$) to examine their separate contributions to the CI.

Step 1: $y=\alpha+\sum_{k} \beta_{k}x_{k}+\varepsilon$ (regression)

Step 2: $CI=\sum_{k} \left( \frac{\beta_{k}\bar{x}_{k}}{u} \right){CI}_{k}+\delta=\sum_{k} \eta_{k}{CI}_{k}+\delta$

$\beta_{k}$, $\bar{x}_{k}$, and ${CI}_{k}$ were the coefficient, mean, and CI of $x_{k}$ (measured the income-related inequalities in $x_{k}$), respectively; $u$ was the mean of $y$, and $\delta$ was the residual. Given the binary diabetes status, we applied probit regression with marginal effects ($\beta_{k}$).

The contribution of $x_{k}$ to the CI was calculated as $\eta_{k}{CI}_{k}/CI$, with a larger value representing a larger contribution.

**3. Examine the contributions of determining factors to the changes in overall CI over time.**

$$\Delta CI={CI}_{2}-{CI}_{1}=\sum_{k} \eta_{k2}\left( {CI}_{k2}-{CI}_{k1} \right)+\sum_{k} {CI}_{k1}(\eta_{k2}-\eta_{k1})+\Delta\delta$$

$\Delta CI$ was the difference in CI between two years. (i.e., $\Delta CI={CI}_{2011}-{CI}_{2001}$ during 2001−2011; $\Delta CI={CI}_{2018}-{CI}_{2011}$ during 2011−2018)

Explanatory variables ($x_{k}$)

Outcome ($y$)

**Demographics**

- Age
- Sex
- Race/Ethnicity

**Socioeconomic status**

- Income

**Other selected factors**

- BMI
- Physical activity
- Smoking
- Health insurance status
- General health status

Diabetes status

- Estimate the CI overall and by sex, age, and race/ethnicity annually in 2001-2018.
- Identify the trend in overall CI using Joinpoint regression, and the turning point is 2011.

In Step 1

**Figure S9 -** Conceptual framework and variable explanations for the analytic approach.

**Reference**

Koolman, X., & Van Doorslaer, E. (2004). On the interpretation of a concentration index of inequality. *Health economics,* 13, 649-656.

Wagstaff, A. (2005). The bounds of the concentration index when the variable of interest is binary, with an application to immunization inequality. *Health economics,* 14, 429-432.

Wagstaff, A., van Doorslaer, E., & Watanabe, N. (2003). On decomposing the causes of health sector inequalities with an application to malnutrition inequalities in Vietnam. *Journal of Econometrics,* 112, 207-223.
